# The anti‐inflammatory effect of metformin: The molecular targets

**DOI:** 10.1111/gtc.13098

**Published:** 2024-02-04

**Authors:** Natsumi Sakata

**Affiliations:** ^1^ School of Medicine Tohoku University Sendai Japan

**Keywords:** cardiovascular disease, cytokine, diabetes, inflammation, metformin

## Abstract

Metformin is an anti‐diabetic drug. Metformin mainly inhibits gluconeogenesis in the liver and reduces blood sugar. In addition to the anti‐diabetic effects, many studies have revealed that metformin has anti‐inflammatory effects. Various molecules were suggested to be the target of the metformin's anti‐inflammatory effects. However, the conclusion is not clear. Metformin is related to a number of molecules and the identification of the main target in anti‐inflammatory effects leads to the understanding of inflammation and metformin. In this article, I discuss each suggested molecule, involved mechanisms, and their relationship with various diseases.

## Graphical abstract

Variable molecules were suggested to be the target of the anti‐inflammatory effects of metformin; however, the conclusion is not clear. In this article, I discuss each molecule, suggested mechanisms, and their relationship with variable diseases.

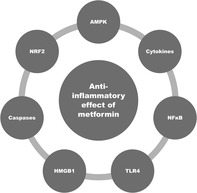

## 1 INTRODUCTION

Metformin is an anti‐diabetic drug used as the first line drug (Flory & Lipska, 2019). Metformin inhibits gluconeogenesis in the liver, and this reduces the blood sugar levels (Rena et al., 2017). In addition, metformin reduces cardiovascular events (Rena et al., 2017; UKPDS group, 1998) and has renoprotective effect (Koroglu‐Aydin et al., 2021). It was also reported that metformin prolongs the life span in *Caenorhabditis elegans* (Chen et al., 2017). Metformin is a multi‐functional drug and metformin use will increase in treatment and prevention of many kinds of diseases.

Metformin regulates multiple cell functions: cell proliferation (Della Corte et al., 2016; Wheaton et al., 2014), glucose metabolism (Argaud et al., 1993; Foretz et al., 2010), lipid metabolism (Luo et al., 2017; Zhou et al., 2001), mitochondrial function (Soberanes et al., 2019; Wheaton et al., 2014), and inflammation (Bharath et al., 2020; Horiuchi et al., 2017; Kang et al., 2014; Tsoyi et al., 2011). This multiple regulation may contribute to the multiple effects of metformin in whole body as mentioned above.

Diabetes accompanies various pathologies: insulin resistance, vascular damage followed by cardiovascular diseases, retinopathy, and chronic kidney disease. Clinical studies indicated that metformin in human body reduces gluconeogenesis in the liver and also insulin resistance (Cree‐Green et al., 2019; Malin et al., 2012). In this several decades, many studies revealed that the inflammation is critical in the progression of diabetes (Borst, 2004; Forrester et al., 2020; J. C. Jha et al., 2022; Karam et al., 2017; Senn et al., 2002; Sharif et al., 2021; Winiarska et al., 2021). Around the same time, it was shown that metformin has anti‐inflammatory effects (Kim et al., 2014; Volarevic et al., 2015; Yuan et al., 2012). Insulin resistance is a key metabolic change in diabetes and is tightly related to chronic inflammation (Rohm et al., 2022; Saltiel & Olefsky, 2017). Inhibition of chronic inflammation is one of the strategies taken to reduce insulin resistance in diabetes. The inflammatory pathways via TNFα, TLR4, NFκB, and NLRP3, improves insulin resistance (Akash et al., 2018; Arkan et al., 2005; Moller, 2000; Shi et al., 2006; Vandanmagsar et al., 2011) and these pathways are the targets of metformin which will be mentioned in following sections. Taken together, metformin may ameliorate diabetes in two ways: regulation of glucose metabolism in liver and inhibition of inflammation.

Many studies have tried to determine the molecular targets of metformin and to reveal the anti‐inflammatory mechanisms. However, we do not know which molecule is the major and critical target of metformin. When we identify the most important target of metformin, we could use metformin and its derivatives as a more selective and effective anti‐inflammatory drug. Comprehensive understanding and comparison of the target molecules are necessary.

## 2 TARGET MOLECULES

The molecules mentioned in this section are shown in Table 1.

**TABLE 1:** Target molecules of metformin in inflammation.

Molecules	Direct interaction with metformin	Related diseases and pathologies except INFLAMMATION	Reference
TNFα	No	Insulin resistance	Tsoyi et al. (2011), Horiuchi and Sakata et al. (2017)
IL‐6	No	Immunity	Tsoyi et al. (2011), Kim et al. (2014). Xiong et al. (2021)
TGFβ	No	Fibrosis of blood vessel	Cheng et al (2016). Chiang et al. (2017)
NF‐κB	Predicted	Chronic inflammatory disease, arteriosclerosis	Isoda et al. (2006), Chiang et al. (2017), Alzokaky et al. (2023)
HMGB1	Yes	Cell damage, arteriosclerosis	Tsoyi et al. (2011), Huang et al. (2018), Horiuchi and Sakata et al. (2017)
TLR4	No	Cell damage, infection	Isoda et al. (2006), Chiang et al. (2017), Horiuchi and Sakata et al. (2017)
Caspase‐3	Predicted	Apoptosis, cell migration	Yuan et al. (2012), Mxinwa et al. (2022), Alzokaky et al. (2023)
Caspase‐8	No	Apoptosis, cell migration	Yuan et al. 2012
NRF2	No	Cardiovascular disease, neurodegenerative disease	Dwivedi et al (2023)
AMPK	Yes	Metabolism, cell growh	Zhou et al. (2011), Kalender et al. 2010, Zhang et al. (2012)

### 2.1 Cytokines

Cytokines are important molecules in inflammatory response and are recently focused as the therapeutic target for various diseases (Fajgenbaum & June, 2020; Lippitz, 2013; Propper & Balkwill, 2022). Therefore, cytokine targeting therapy has been focused on as a novel therapy for various diseases: cancer (Berraondo et al., 2019; Dougan et al., 2018), heart failure (Hanna & Frangogiannis, 2020), rheumatoid arthritis (RA) (Noack & Miossec, 2017), and lymphohistiocytosis (H. Q. Zhang et al., 2022).

Tumor necrosis factor α (TNFα) is one of the critical cytokines in many kinds of disease, such as RA (D. I. Jang et al., 2021). A study using mice reported that TNFα is related to the renoprotective effect of metformin (Christensen et al., 2016). Metformin inhibits lipopolysaccharide (LPS)‐induced TNFα production in vitro (Tsoyi et al., 2011) and in vivo (Horiuchi et al., 2017). Since TNFα inhibitors, such as infliximab, etanercept, and adalimumab, are effective in RA and other autoimmune diseases (Lim et al., 2018), metformin's anti‐TNFα function could be effective in these diseases. It is also known that TNFα is related to the insulin resistance in type 2 diabetes (Akash et al., 2018; Moller, 2000). The reduction of TNFα could be a part of the anti‐diabetic mechanism of metformin. However, the direct target of metformin that regulates TNFα production is unknown.

Interleukin‐6 (IL‐6) is a cytokine produced in stromal cells and inflammatory cells that stimulates lymphocytes (Hunter & Jones, 2015). Metformin inhibits IL‐6 expression in macrophages (Kim et al., 2014; Tsoyi et al., 2011; Xiong et al., 2021) and endothelial cells (Isoda et al., 2006) in inflammatory condition. Metformin also inhibits IL‐6‐induced inflammatory response (Cansby et al., 2014; Xiong et al., 2021). Considering these results, metformin regulates IL‐6 function via two mechanisms, inhibition of IL‐6 expression and of its downstream signaling, although whether metformin directly interacts with IL‐6 is unclear.

Transforming growth factor β (TGFβ) is a cytokine that regulates immune response and fibrillation in wound healing process (Morikawa et al., 2016; Travis & Sheppard, 2014). It was shown that metformin inhibits TGFβ expression (Chiang et al., 2017) and TGFβ‐induced cell proliferation as well as migration (Cheng & Hao, 2016). These results could be related to the metformin's cardioprotective and vasoprotective effects because TGFβ is an important factor in fibrosis in various tissues including blood vessels (Chung et al., 2021; Cunha et al., 2017).

Since many kinds of cells, not only inflammatory cells, interact via cytokines, metformin should have the effect on a number of cell types. In other words, metformin's effect is not specific to cell type or cytokine, and this could be both advantage and disadvantage.

### 2.2 Nuclear factor‐κB

Nuclear factor‐κB (NF‐κB) is a key transcriptional factor which is activated by multiple stimuli (Hayden & Ghosh, 2008; Lawrence, 2009) and regulates various inflammatory factors, TNFα, IL‐1β, and IL‐6 and such (Tak & Firestein, 2001). NF‐κB activation is associated with many chronic inflammatory diseases: ulcerative colitis, multiple sclerosis, and atherosclerosis (Tak & Firestein, 2001). Several studies revealed that metformin targets NF‐κB pathway to inhibit inflammation (Chiang et al., 2017; Isoda et al., 2006). In addition, a recent study suggested that metformin directory binds NF‐κB (Alzokaky et al., 2023). As mentioned above, metformin regulates various inflammatory factors including NF‐κB regulated factors. Considering these findings, NF‐κB may be a key target of metformin. This means metformin might be effective on chronic inflammatory diseases mentioned above.

### 2.3 High mobility group box 1

High mobility group box 1 (HMGB1) is a multi‐functional protein. HMGB1 functions in both intracellular and extracellular space (Kang et al., 2014). HMGB1 localizes mainly in the nucleus. However, in some conditions, HMGB1 is released to extracellular space passively from damaged cells (Venereau et al., 2016) and actively from activated inflammatory cells (Bonaldi et al., 2003). Released HMGB1 is a kind of damage‐associated molecular patterns (DAMPs) and functions like cytokine and chemokine to induce inflammatory responses (Rubartelli & Lotze, 2007; Venereau et al., 2015). Also, the extracellular HMGB1 is a ligand of TLR4 (mentioned in the following section). It has been revealed that metformin inhibits inflammation via HMGB1 in three ways: inhibition of expression (Huang et al., 2018), reduction of release from macrophage cells (Tsoyi et al., 2011), and direct binding to extracellular HMGB1 (Alzokaky et al., 2023; Horiuchi et al., 2017). HMGB1 is a sole extracellular factor that the direct binding is validated by biochemical analysis (Horiuchi et al., 2017).

### 2.4 Toll‐like receptor 4

Toll‐like receptor 4 (TLR4) is one of the pattern recognition receptors expressed by inflammatory cells like macrophages and neutrophils. TLR4 activation induces inflammatory response especially against the infection of bacteria, viruses, and fungus (Novus Biologicals, 2014). TLR4 recognizes LPS, a component of Gram‐negative bacteria, and some kinds of DAMPs: HMGB1, S100 calcium‐binding protein A4 (S100A4), HSPB5 and such (Bolourani et al., 2021; Mzyk et al., 2022). DAMPs are intracellular molecules that are released to extracellular space from the damaged cells. Thus, TLR4 recognizes both exogenous and endogenous inflammatory factors. TLR4 induces the activation or transcription of the metformin targets mentioned above: NFκB (A. K. Jha et al., 2021; Takeuchi & Akira, 2010), TNFα (Caldwell et al., 2014; Rusai et al., 2010), IL‐6 (Abou‐Hany et al., 2018; A. K. Jha et al., 2021), and TGFβ (Seki et al., 2007). In addition, HMGB1, a direct target of metformin, is an endogenous ligand of TLR4 (Alzokaky et al., 2023; Horiuchi et al., 2017). These results are suggesting the relationship between metformin and TLR4 signaling pathway via downstream factors. In other words, TLR4 pathway includes many factors which regulates inflammation and metformin works as TLR4 pathway inhibitor. This may be a reason why many factors were identified as metformin targets.

### 2.5 Caspases

Caspase is a cysteine protease family involved in cell cycle, cell differentiation, apoptosis, and inflammation (Eskandari & Eaves, 2022; Van Opdenbosch & Lamkanfi, 2019). Many factors, like pathogen, DAMPs, and cytokines activate the caspases and induce inflammation and cell death (Van Opdenbosch & Lamkanfi, 2019). Especially caspase‐3, ‐7, and ‐8 are important in inflammatory response (Newton et al., 2021). It was shown that metformin inhibited the activity of caspase‐3 and ‐8 in LPS‐induced inflammation model (Yuan et al., 2012) and the expression of caspase‐3 in lymphocytes (Mxinwa et al., 2022). In addition, a computer prediction suggested that metformin directly binds caspase‐3 (Alzokaky et al., 2023). Metformin's apoptosis regulation may have both pathways, caspase‐dependent and ‐independent (J. H. Jang et al., 2020; Rosidi et al., 2023; Zheng et al., 2020). The interaction between caspase family and metformin could be the mechanism behind multiple functions of metformin.

### 2.6 Nuclear factor erythroid 2‐related factor 2

The oxidative stress is tightly related to the inflammation and aggravates cardiovascular disease (Guzik & Touyz, 2017; Papaconstantinou, 2019), sepsis (Joffre & Hellman, 2021), nonalcoholic fatty liver disease (NAFLD) (Paradies et al., 2014), cancer (Tsang et al., 2022), and neurodegenerative disease (Chen & Zhong, 2014; Sumien et al., 2021). Nuclear factor erythroid 2‐related factor 2 (NRF2) is a transcriptional factor and targets antioxidant genes and has an anti‐inflammatory function (Saha et al., 2020). In NAFLD model mice, metformin increased NRF2 and NRF2 target gene expression in the liver (Dwivedi & Jena, 2023). The molecular mechanism and interaction between metformin and NRF2 are unclear and the function of metformin in the response to the oxidative stress should be studied more.

### 2.7 AMPK

AMPK is the most investigated metformin target (Agius et al., 2020; Zhou et al., 2001). Metformin activates AMPK by direct binding (Y. Zhang et al., 2012). However, later studies indicated that metformin functions both AMPK‐dependently (Foretz et al., 2010; Zhou et al., 2001) and AMPK‐independently (Ben Sahra et al., 2011; Deschemin et al., 2017; Kalender et al., 2010; Moonira et al., 2020). Other studies showed that metformin activates AMPK by increasing adenosine monophosphate/adenosine triphosphate (AMP/ATP) ratio (Foretz et al., 2010; Stephenne et al., 2011). AMPK modifies the general and many kinds of cell functions, like metabolism, cell growth, and inflammation (Garcia & Shaw, 2017; Trefts & Shaw, 2021). It is true that AMPK is a major metformin's target, but it is difficult to determine whether AMPK is an inflammation‐specific target or not.

## 3 CONCLUSION

Metformin has the anti‐inflammatory effects and is beneficial in the treatment of various diseases. One of the metformin's anti‐diabetic mechanisms is the inhibition of gluconeogenesis in the liver and it is considered that metformin's main target organ is the liver (Agius et al., 2020; Flory & Lipska, 2019). This may be the reason why many studies use the liver inflammation model to reveal the anti‐inflammatory mechanism of metformin (Hadi et al., 2012; Horiuchi et al., 2017; Poon et al., 2003; Yuan et al., 2012). This is reasonable but we need much more studies about metformin in other organs: kidney, blood vessels, muscles, and so on, especially the organs related to the insulin resistance. It is well known that the blood vessel in diabetic patients is damaged by chronic inflammation (Kenny & Abel, 2019; Li et al., 2023; Wang et al., 2020). The reduction of inflammation in blood vessel could be the mechanism of cardioprotective effects of metformin.

A number of molecules are suggested to be a target of metformin (Table 1). It is still unclear which molecule is the most critical. Horiuchi and Sakata et al. (2017) suggested that metformin binds negative charge domain in HMGB1 by its positive charge. However, no study has verified this. In addition, no study has shown the structure of metformin‐target complex obtained by X‐ray crystallography or cryo‐electron microscopy. Therefore, the protein structure or the amino acid sequence which is common and specific for metformin interaction is unknown. It seems that metformin interacts with various proteins with low specificity. It is reasonable to find out whether molecules other than proteins, such as nucleotide, lipid, and carbohydrate can be the direct target of metformin.
